# Around the Hospitals

**Published:** 1894-09-08

**Authors:** 


### Around the Hospitals.

[Notice to the Managers of Institutions.—Special space is now reserved for the insertion of notices of hospital meetings and festivals. The Editor requests that all notices may reach The Hospital Office, 428, Strand, at least one week before the date of each meeting.]

**The Huddersfield Infirmary.**—The 63rd annual report of this hospital has just been issued, and from a pecuniary point of view is distinctly satisfactory. To read that "notwithstanding the continued depression in trade the income has about equalled the expenditure," is encouraging, especially as the report shows that the committee are contemplating certain necessary improvements and alterations. Some falling off is noticed in the workpeople's contributions, which amount to £860 6s. 1d., a decrease of £118 4s. 7d. compared with last year, but the committee attribute this decrease entirely to bad times. The proposed improvements are intended to be as thorough as possible, in order to ensure a result completely satisfactory. The cost is estimated at about £15,000, and the board are glad to be able to announce that of that sum no less than £12,000 had been already promised. No doubt the remaining balance will quickly be forthcoming, since the people of Huddersfield are evidently inclined to support their medical charities well. The convalescent home at Meltham Mills fully maintains its previous satisfactory condition.

**The Princess Alice Memorial Hospital, Eastbourne.**—This hospital shows a deficiency on the accounts of last year, but it is a very small one. The management have to congratulate themselves that the debt on the building of the children's ward has been cleared off during the same period. A larger number of patients were received than in the previous year, and this accounts for the extra expenditure. The annual subscriptions show a happy increase. In spite of the usual losses to be expected amongst subscribers, forty-eight new subscribers have been placed on the books. A welcome addition to the endowment fund was made during the year by an anonymous donation of £200. The hospital adopts the wise course of requiring payment to be made by patients when able to do so. The average payment per patient was 8s. 4d. per head, a lower sum than has ever accrued to the hospital, owing to the poverty of those who were treated. The total number of cases treated in the hospital during the year was 305. The income amounted to £1,500; the expenditure to £1,598.

